# Sarcoidosis and Pain Caused by Small-Fiber Neuropathy

**DOI:** 10.1155/2012/256024

**Published:** 2012-12-05

**Authors:** Lara Heij, Albert Dahan, Elske Hoitsma

**Affiliations:** ^1^Department of Anesthesiology, Leiden University Medical Center, P5Q, Postbus 9600, 2300 RC Leiden, The Netherlands; ^2^Department of Neurology, Diaconessenhuis Leiden, Leiden, The Netherlands

## Abstract

Sarcoidosis is a chronic inflammatory illness and small-fiber neuropathy (SFN) is one of the disabling and often chronic manifestations of the disease. SFN presents with peripheral pain and symptoms of autonomic dysfunction. The character of the pain can be burning or shooting. Besides, allodynia and hyperesthesia can exist. Diagnosis is usually made on the basis of clinical features, in combination with abnormal specialized tests. The aim of treatment is often to reduce pain; however, total pain relieve is seldom achieved. The role of TNF-**α** in the pathogenesis of SFN in sarcoidosis appears interesting to explore. Novel therapeutic agents such as ARA 290, a nonhematopoietic erythropoietin analogue with potent anti-inflammatory and tissue protective properties, are interesting to explore in the treatment of SFN in sarcoidosis.

## 1. Sarcoidosis

**Figure 1:**
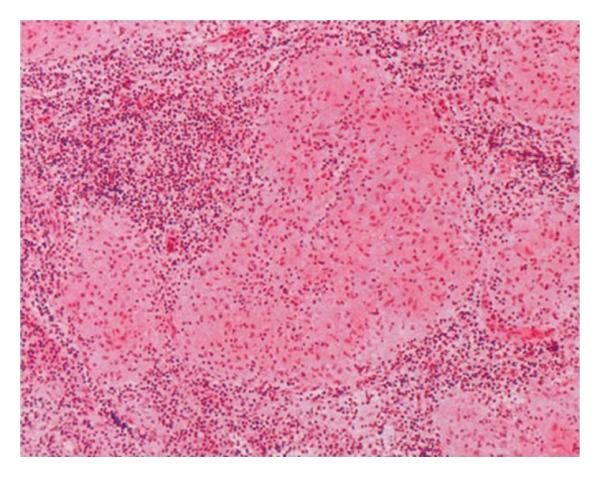
A microscopical section of mediastinal lymph node with HE stain, ×40. Multiple granulomas with various sizes from 0,2 to 0,8 mm in diameter are observed in the lymph node. These granulomas consist of histiocytes, which have large cytoplasm and partly connect to each other but lack a necrotic region.

Sarcoidosis has been known for more than 100 years and has been first described by the dermatologist Hutchinson and several years later by two other dermatologists, Besnier and Boeck. It is a multiorgan inflammatory disorder that is characterized by noncaseating granuloma (Figure 1). The exact etiology remains unknown. It is suspected that exposure to one or more extrinsic antigens in a genetically susceptible individual leads to the overactivation of inflammatory pathways that promote the formation of sarcoid granuloma [1]. Granuloma formation is regulated by a complex interaction between T-helper lymphocytes and macrophages, in which cytokines such as tumor necrosis factor (TNF)-*α* play an important role.

The clinical course of sarcoidosis is highly variable and depends on ethnicity, duration of illness, site and extension of organ involvement, and activity of the granulomatous process, which shows a tendency to wax and wane. Mode of presentation varies from asymptomatic, to an “acute onset” presenting as Lofgren's syndrome and to a chronic course, frequently accompanied with pain and fatigue. Practically every organ can be involved. However, most commonly (>90%) the lungs are affected [2, 3]. Often patients suffer from symptoms a long time before the diagnosis sarcoidosis is confirmed. Due to the manifold presentation of the disease, it is a challenge to recognize in an early phase. The acute stage of disease usually presents itself with erythema nodosum, arthritis, fever, and fatigue with a good prognosis. Spontaneous remission usually occurs within two years, while chronic sarcoidosis mostly has an insidious onset with often relapses, resolution being less likely. In some of the cases, the disease is progressive. Development of lung fibrosis, cardiac sarcoidosis, and neurosarcoidosis is related to worse prognosis. Factors that trigger the formation of fibrosis in sarcoidosis are poorly understood. Up to 5% will eventually die from sarcoidosis. 

In chronic sarcoidosis, pain and fatigue are important symptoms, even when sarcoidosis is clinically in remission fatigue and pain may persist and become a chronic complaint. These complaints often result in a severe reduction in quality of life. Although a lot of research has been done, the exact mechanism behind this “postsarcoidosis chronic fatigue syndrome” remains unsolved. 

Recently, it has been shown that pain in patients with sarcoidosis is often related to neuropathy of small fibers of the peripheral nervous system [4–7]. 

## 2. Small Fiber Neuropathy

Small-fiber neuropathy (SFN) is a peripheral nerve disorder that selectively affects thinly myelinated A*δ* fibers and unmyelinated C fibers. Small nerve fibers are involved in both somatic and autonomic function [8]. As a result, patients with SFN may present with symptoms of neuropathic pain (NP) and autonomic dysfunction [5]. 

Damage to or loss of small somatic nerve fibers results in burning pain, tingling, or numbness that typically affects the limbs in a distal to proximal gradient. Symptoms are usually worse at night and often affect sleep. People sometimes sleep with the feet uncovered because they can not bear the touch of the sheets. Besides, walking may be difficult due to pain by the pressure on the floor. When autonomic fibers are affected, patients may experience dry eyes, dry mouth, orthostatic dizziness, constipation, bladder incontinence, sexual dysfunction, hyperhidrosis or hypohidrosis, or red or white skin discoloration. Finally restless legs syndrome may be present, characterized by disagreeable leg sensations that usually occur prior to sleep onset and cause an almost irresistible urge to move (Table 1).

Most patients suffer from length-dependent small-fiber neuropathy (LD-SFSN): symptoms and signs start to develop in the toes and feet, symptoms gradually progress to involve distal legs, fingertips, and hands. Non-length-dependent small-fiber neuropathy (NLD-SFSN) is not as common as LD-SFSN and patients develop complaints in a patchy distribution. This can include face, upper limbs, or trunk before the lower limbs are affected. The NLD-SFSN is more seen in women and presents at a younger age [9, 10]. 

### 2.1. Diagnosis of Small Fiber Neuropathy

Nerve conduction studies, which are the key in evaluation of other (large fiber) neuropathies, are generally normal in SFN. Therefore, the syndrome of SFN has been an enigma to practitioners because of the unexplained contrast between severe pain and a paucity of neurological and electrophysiological findings. Recent advantages in diagnostic techniques facilitate objective confirmation of clinical diagnosis and characterization of fiber involvement. However, a golden standard for the diagnosis of SFN is not available yet. Diagnosis is usually made on the basis of clinical features, in combination with abnormal specialized tests, which include among others, assessment of intraepidermal nerve fiber density (IENFD) in skin biopsy, temperature sensation tests, and sudomotor and cardiovagal testing for autonomic fibers [4, 6, 11]. However, all tests have their limitations. 

Quantitive sensory testing (QST) includes temperature threshold testing. Thermal (cold and warm) and mechanical (tactile and vibration) detection thresholds assess small-fiber function (including the central pathways). The cold detection threshold (CDT) examines the A-delta-fiber function, while assessment of C-fibre function is examined by the warm detection threshold (WDT). The major limitation of QST is its psychophysical character. As a consequence, malingering and other nonorganic factors can influence test results [12]. 

To objectively test small nerve fibers, laser-evoked potentials (LEP) and contact heat-evoked potentials (CHEPs), have been developed. It is well established that both laser and contact heat stimulation activate thermo-nociceptive cutaneous nerves. Even though attention and other cognitive processes influence the amplitude of Laser Evoked Potentials (LEPs) and Contact Heat Evoked Potentials (CHEPs) these tests carry up relevant information on the functional state of nociceptive terminals.

For the CHEPs, a thermofoil thermode stimulator is used to reach a temperature of 53°C at a rate of 70°C/s. It has been shown that patients with sensory neuropathy have lower-amplitude CHEPs, which correlates with other SFN tests [13].

**Figure 2:**
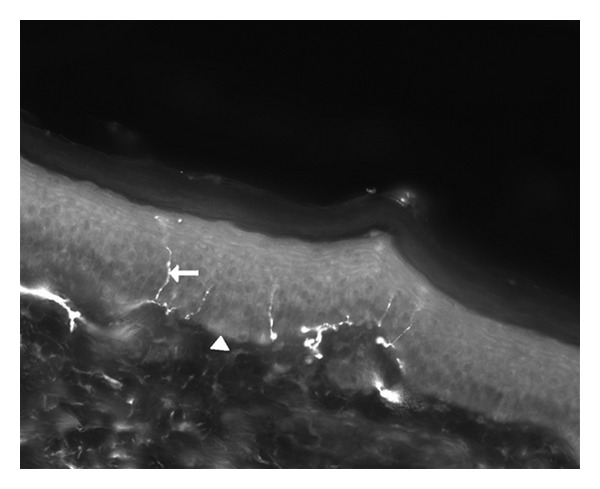
Magnification 200x. Punch skin biopsy from a healthy control showing intraepidermal nerve fibers. Arrow: intraepidermal nerve fiber. Arrowhead: basal membrane (above the basal membrane the epidermis is shown, under the basal membrane the dermis is shown).

Multiple studies have emphasized the importance of intraepidermal nerve fiber density (IENFD) assessment using PGP-9.5 immunofluorescent staining in skin biopsy in the evaluation SFN [14]. Epidermal nerves are the distal terminals of small dorsal root ganglia neurons that pierce the dermal-epidermal basement membrane and penetrate the epidermis. The discovery of the antibody to the neuropeptide protein gene product (PGP) 9.5 made it possible to effectively stain nerve fibers (Figure 2). PGP 9.5 is a ubiquitin C-terminal hydrolase and is enriched in epidermal nerve fibers [14]. A punch biopsy is performed following established procedures, mostly 10 cm above the lateral malleolus after local anesthesia with 1% lidocaine. A limitation of skin biopsies is that they are available in only a few academic centers. The histological technique is moderately complicated and time consuming, and before implementing it, a relatively large subset of healthy controls should be studied as the normative range is wide. Finally skin biopsy appears to have a high specificity but low sensitivity in sarcoidosis: Bakkers in 2009 showed that 32,8% of sarcoidosis patients with symptoms of SFN had a reduced IENFD score in the skin biopsy, and 14,3% in patients without SFN symptoms had a reduced IENFD [4]. The rule “physicians, not tests make diagnosis” appears especially applicable for SFN. Examination often reveals allodynia, hyperalgesia, or reduced pinprick and thermal sensation in the affected area. Motor strength en proprioception, however, are (as functions of the large fibers) preserved. 

### 2.2. Etiology of Small Fiber Neuropathy

In 50% of the cases presenting with SFN no underlying disease is found: “idiopathic SFN” [15]. Recent studies have shown gain of function mutations in sodium channel Na(V)1.7 in a subset (28.6%) of those patients with idiopathic SFN [16]. The exact role of these mutations is unresolved yet. 

In 50% of the cases presenting with SFN, an underlying disease is present, including diabetes, sarcoidosis, and amyloidosis among others (Table 2) [6]. It is remarkable that SFN appears frequent in several immune-mediated diseases. This leads to the hypothesis that there might be a common pathway in immune-mediated diseases resulting in SFN. The idea of an immune-mediated mechanism as the cause of SFN has also been reported by others [17–19].

The pathogenetic role of oxidative stress, inflammatory cytokines such as TNF-*α*, and neuropeptides such as substance P (SP) are interesting to explore as a common final pathway in SFN in several immune-mediated inflammatory diseases. We described a patient with severe SFN who showed spectacular improvement after treatment with anti-TNF-*α* therapy [20]. This case supports the idea that TNF-*α* may be a crucial cytokine in the pathogenesis of SFN related to sarcoidosis and presumably in SFN related to other immune-mediated inflammatory diseases as well. Theoretical support for the effect of anti-TNF-*α* therapy on SFN may be found in the following. First, TNF-*α* plays an important role in immune-mediated neuropathies such as Guillain-Barré syndrome, in which small nerve fibers are also involved. Elevated serum concentration of TNF-*α* shows a positive correlation with neuropathy severity in patients with Guillain-Barré syndrome. Furthermore, the decrease in serum TNF-*α* and increase in serum soluble TNF receptors show a positive correlation with neuropathy recovery in those patients. Second, pharmacological and physiological studies report that proinflammatory cytokines such as TNF-*α* are strongly involved in the generation and maintenance of neuropathic pain [21–25].

## 3. Treatment

Although alternatives to corticosteroids have been frequently administered in this disease, corticosteroids remain the mainstay of treatment in sarcoidosis. Immunosuppressive agents (chlorambucil, cyclophosphamide, methotrexate, cyclosporine, azathioprine), anticytokine agents (thalidomide, pentoxifylline), antimalarials (chloroquine, hydroxychloroquine), melatonin, and monoclonal antibody (infliximab) have been used in chronic resistant sarcoidosis [26]. 

Usual treatments in sarcoidosis such as prednisone and methotrexate do not appear beneficial in sarcoidosis-related SFN (personal experience). SFN is disabling for patients and the pain is often difficult to treat. SFN has a high impact on the quality of life and often invalidates the patient. Case reports mention beneficial effects of intravenous immunoglobulin [19] and anti-TNF-alpha therapy [20]. The exact potency of these drugs needs further study, however. 

Symptomatic neuropathic pain treatment in sarcoidosis patients is not different from treatment of neuropathic pain from other causes and consists of antidepressants, anticonvulsants and prolonged-release opioids (Table 3). However, in common with their effects in other neuropathic pain states, these agents provide limited pain relief in just 30–60% of patients, at the cost of considerable side effects. These data indicate that there is an imminent need for analgesic agents with high efficacy in neuropathic pain patients without causing debilitating side effects.

## 4. Directions for Future Studies

As the role of TNF-*α* in the pathogenesis of SFN in sarcoidosis appears interesting to explore, anti-TNF therapy might be beneficial in the treatment of SFN in sarcoidosis. A recent therapeutic development has been the availability of agents that directly inactivate the proinflammatory cytokine TNF-*α*. Those are expensive drugs with possible severe side effects including opportunistic infection. 

Recently, we initiated a program aimed at the treatment of neuropathic pain in patients with sarcoidosis with a novel therapeutic agent, ARA 290. ARA 290 is a nonhematopoietic erythropoietin analogue with potent anti-inflammatory and tissue protective properties, acting at the innate repair receptor [27–29]. In recent years, an endogenous system has been identified that antagonizes the production and action of proinflammatory cytokines that are involved in promoting tissue injury, while simultaneously activating repair processes. The primary mediator of this system is hypoglycosylated erythropoietin (EPO) that acts through a unique receptor isoform, the innate repair receptor (IRR), which is a combination of EPO and beta common receptor subunits. Many diverse preclinical models of tissue injury have demonstrated the efficacy of EPO as an effective cytoprotectant and activator of healing and repair. For example, EPO acting through the IRR has been shown to improve recovery and function from nerve injury in a variety of preclinical models, including the small-fiber neuropathy caused by uncontrolled diabetes [28]. Because the IRR has a lower affinity for EPO than the receptor utilized in hematopoiesis (~2*·*0–20*·*0 nM versus 0*·*2 nM resp.), larger doses of erythropoietin must be administered to activate the IRR. Since EPO interacts with both of these receptors, translation of this knowledge into clinical use has been hindered by the presence of unavoidable hematopoietic side effects triggered by the hematopoietic receptor. For example, clinical studies evaluating use of EPO for tissue protection have consistently revealed increased rates of serious thrombosis [28]. To circumvent this problem, a number of IRR-specific ligands have been engineered. 

One novel approach is pyroglutamate helix B surface peptide (ARA 290). This peptide mimics the spatial configuration of EPO that is believed to interact with the IRR. In spite of having a plasma half life of less than 2 minutes, ARA 290 is as efficacious as EPO in a wide variety of models of tissue injury. Additionally, preclinical toxicology studies of ARA 290 and single- and multiple-ascending repeated dosing of human volunteers and patients with kidney disease, diabetes mellitus, or sarcoidosis have raised no safety issues (unpublished data, Araim Pharmaceuticals).

First studies in animals (with nerve-damage induced neuropathic pain) and in patients with chronic neuropathic pain from sarcoidosis and diabetes mellitus indicated that ARA 290 is highly effective in causing pain relief in these neuropathic pain states. This compound appears potential for this chronic inflammatory disease and further investigation has been started.

## Figures and Tables

**Table 1:** Symptoms of small fiber neuropathy.

Sensory symptoms	Pain*
	Paraesthesias
	Sheet intolerance
	Restless legs syndrome**
Symptoms of autonomic dysfunction	Hypo- or hyperhidrosis
	Diarrhoea or constipation
	Urinary incontinence or -retention
	Gastroparesis
	Sicca syndrome
	Blurry vision
	Facial flushes
	Orthostatic intolerance
	Sexual dysfunction

*Pain in small fiber neuropathy is often burning, tingling, shooting, or prickling in character.

**Restless legs syndrome is a disorder characterized by disagreeable leg sensations that usually occur prior to sleep onset and that cause an almost irresistible urge to move.

**Table 2:** Causes of small fiber neuropathy [6].

Idiopathic	
	Familial amyloidosis
	Autosomal recessive hereditary neuropathy
	Hereditary sensory and autonomic neuropathy
Inherited	Fabry's disease
	Ross syndrome
	Friedreich's ataxia
	Tangier disease
	Diabetes mellitus
	Impaired glucose tolerance
	Alcoholism
	Systemic amyloidosis
	Vasculitis
	Sarcoidosis
	Sjögren's disease
Acquired	Systemic lupus erythematosus
	Guillain-Barre syndrome
	Antecedent viral infection
	HIV
	Antisulfatide antibodies
	Hyperlipidemia
	Complex regional pain syndrome
	Paraneoplastic syndrome
	Neurotoxic medication

**Table 3:** Drugs for pain control in small fiber neuropathy.

Drug	Dosage (per day)	Common side effects
Antidepressants		Sedation, weight gain, anticholinergic effects, sexual dysfunction, arrhythmia (side effects most prominent Sedation, weight gain, anticholinergic effects, sexual dysfunction, arrhythmia (side effects most prominent with amitriptyline)
Amitriptyline (Elavil)	20–150 mg	
Nortriptyline (Aventyl)	20–150 mg	
Desipramine (Norpramin)	20–200 mg	
Duloxetine (Cymbalta)	60–120 mg	
Anticonvulsants		
Gabapentin (Neurontin)	600–3,600 mg	Sedation, dizziness, peripheral edema, weight gain
Pregabalin (Lyrica)	150–600 mg	Similar to gabapentin
Topiramate (Topamax)	25–400 mg	Weight loss, sedation, cognitive slowing, renal stones, paresthesias
Lamotrigine (Lamictal)	25–400 mg	Stevens-Johnson syndrome, rash, dizziness, nausea, sedation
Carbamazepine (Tegretol)	200–1,200 mg	Dizziness, sedation, ataxia, aplastic anemia, liver enzyme elevation
Oxcarbazepine (Trileptal)	600–2,400 mg	Dizziness, nausea, fatigue, leukopenia
Topical anesthetics		
5% Lidocaine patch (Lidoderm)	Every 12 hours	Local edema, burning, erythema
0.075% Capsaicin patch	Three or four times a day	Burning
Opioids, opioid agonists		
Tramadol (Ultram)	100–400 mg	Sedation, dizziness, seizures, nausea, constipation
Oxycodone (Oxycontin)	10–100 mg	Sedation, constipation, nausea; potential for addiction and abuse

## Abbreviations

SFN: Small-fiber neuropathy
TNF-*α*: Tumor necrosis factor-*α*
NP: Neuropathic pain
LD-SFSN: length dependent small-fiber neuropathy
NLD-SFSN: Non-length-dependent small-fiber neuropathy
IENFD: Intra-epidermal nerve fiber density
QST: Quantitive sensory testing
CDT: Cold detection threshold
WDT: Warm detection threshold
PHS: Paradoxical heat sensation
MDT: Mechanical detection threshold
VDT: Vibration detection threshold
CPT: Cold pain threshold
HPT: Heat pain threshold
PPT: Pain pressure threshold
MPT: Mechanical pain threshold
LEP: Laser evoked potential
CHEPs: Contact heat evoked potentials.
